# The 19-Item Environmental Knowledge Test (EKT-19): A short, psychometrically robust measure of environmental knowledge

**DOI:** 10.1016/j.heliyon.2023.e17862

**Published:** 2023-06-30

**Authors:** Lois Player, Paul H.P. Hanel, Lorraine Whitmarsh, Punit Shah

**Affiliations:** a Department of Psychology, University of Bath, Bath, BA2 7AY, UK; b Department of Psychology, University of Essex, Colchester, CO4 3SQ, UK; c Centre for Climate Change and Social Transformations, Cardiff University, Cardiff, CF10 3AT, UK

**Keywords:** Environmental knowledge, Factor analysis, Item response theory, Psychometrics, Self-report

## Abstract

Environmental knowledge is considered an important pre-cursor to pro-environmental behaviour. Though several tools have been designed to measure environmental knowledge, there remains no concise, psychometrically grounded measure. We validated an existing measure in a British sample, confirming that it had good one- and three-factor structures in line with previous literature. For the first time in this field, we built upon previous Classical Test Theory approaches and used discrimination values derived from Item Response Theory to select the best items, resulting in the 19-Item Environmental Knowledge Test (EKT-19). This measure retained a clear factor structure and had moderate-to-good internal reliability, indicating that it is a parsimonious and psychometrically robust measure for the assessment of overall and specific types of environmental knowledge. The theoretical implications and real-world applications of this measure are discussed.

## 1 Introduction

Environmental agencies commonly seek to foster pro-environmental action by enhancing the public's environmental knowledge [1,2]. Although other variables, such as environmental attitudes and values [3,4], are key to fostering impactful pro-environmental behaviours (e.g., avoiding driving or flying [5]), environmental knowledge remains an important prerequisite to pro-environmental behaviour [6], and other environmental outcomes such as policy support [7]. Importantly, it is a core element of the Value-Belief-Norm (VBN) model of environmental action [8], which posits that pro-environmental behaviour is driven by one's environmental values and knowledge, including one's awareness of environmental problems.

Many tools have been designed to measure environmental knowledge. Early work assessed objective knowledge about environmental issues by asking factual questions about how eco-systems function [[9], [10], [11]], known as ‘system’ knowledge. This work often assumed that environmental knowledge was a singular construct, with a unidimensional structure. More recent work has extended this model to encompass three distinct knowledge types, which are theorised to differentially predict pro-environmental behaviour [12]. Beyond ‘system’ knowledge, these include ‘action-related’ knowledge about the best courses of ecological action, and ‘effectiveness’ knowledge, which determines the relative gain of actions [13].

Despite the widespread use of the three knowledge types when assessing environmental behaviours [3,14,15], most studies have not directly compared three-factor solutions against a parsimonious unidimensional model. This has often resulted in previous work measuring ‘action-related’ knowledge as a distinct outcome from ‘system’ or ‘effectiveness’ knowledge, without sufficient psychometric evidence for this (e.g., Ref. [6]). It is therefore essential to understand if environmental knowledge is better conceptualised as a singular type of knowledge or three separate types, as we do in the current study.

Further, many studies have failed to replicate a three-dimensional structure, arguing that environmental knowledge is instead a unidimensional construct [16]. This ambiguous conceptualisation of environmental knowledge has often resulted in inconsistent measurement, and uncertainty about the relative contribution of knowledge to climate-related outcomes, such as pro-environmental behaviour and policy support. Indeed, some studies have noted a moderate positive relationship between environmental knowledge and pro-environmental behaviour [17], yet some have noted a smaller, cross-sectional relationship [18]. Aiming to address these issues, a recent study by Geiger et al. [19] sought to empirically compare a one- and three-factor structure, and create an updated and objective Environmental Knowledge Test.

### 1.1 The Environmental Knowledge Test

Geiger and colleague's Environmental Knowledge Test (EKT [19]) was developed drawing upon earlier, longer measures (e.g., Refs. [16,20]), as well as environmental education books, curricula, and webpages. Their final 36-item scale spanned seven core environmental topics: ecology, climate, resources, consumption behaviours, society and politics, economics, and environmental contamination, and could be classified according to their knowledge type (21 system, 7 action-related, 8 effectiveness items; see Ref. [13]). At scale level, their final measure was deemed suitably difficult (mean item difficulty = 0.686), with acceptable overall discrimination (biserial item-factor correlation = 0.443) in their German sample. Notably, they found no significant difference between their one- and three-factor models, but concluded that the one-factor solution was best for reasons of parsimony, and had good internal reliability (ω = 0.737).

### 1.2 The need for a shorter measure

Given the potential importance of environmental knowledge to important climate-related outcomes (e.g., pro-environmental behaviour and policy support), it is vital that instruments are not only valid and reliable, but also practical. There have been recent calls for shorter measures of psychological constructs (see Ref. [21]), stemming from concerns about data quality. For example, longer measures have been seen to result in missing data, and lower reliability and validity levels due to participant fatigue and boredom [22], especially when participants are completing a battery of measures in one study [21]. While maintaining data quality, measures are commonly shortened over time (e.g., the Autism-Quotient Short developed from the Autism-Quotient [23,24]).

Currently however, there is no standalone, widely-used, concise measure of environmental knowledge. In some instances, this has resulted in researchers creating ad-hoc measures by selecting items from previous research and environmental education programmes [3,15,25]. Such practices may have contributed to the inconsistent relationships observed between environmental knowledge and climate-orientated behaviour (see Ref. [19] for discussion), and fuelled concerns about the validity of existing measures given their untested psychometric properties (e.g., Refs. [26,27]). Other practical issues, such as questions being culturally-specific [28] have also impeded the development of a well-used, psychometrically robust measure of environmental knowledge. Given these challenges, we sought to validate the EKT's factor structure in a British sample, and adopt a novel approach to create a short, and reliable measure of environmental knowledge.

### 1.3 Extending Classical Test Theory

Geiger et al. [19] determined the suitability of their original items using a Classical Test Theory (CTT) approach. Whilst this method is useful in evaluating the overall reliability and validity of a scale in factor analyses of the items, it is not able to provide specific information about the usefulness of individual items [21]. Further, CTT approaches often result in the creation of longer measures, given that reliability often increases with number of items [29]. Relatedly, CTT results in the validation of items at a test-level, meaning that individual items cannot be removed or used alone, since they have not been independently validated [30].

Increasingly, novel approaches such as Item Response Theory (IRT) are beginning to be used to build upon existing environmental measures, such as in the re-validation of the New Ecological Paradigm (NEP; [31], see Ref. [32]). Broadly, this approach seeks to understand the relationship between a latent trait (e.g., environmental knowledge) and individual test items, by assessing the scale's psychometric properties. This is usually achieved by considering item difficulty, used to describe how difficult it is to achieve a 0.5 probability of a correct response given the respondent's ability level, and item discrimination, defined as the rate at which the probability of endorsing a correct item changes, given ability levels. In contrast to CTT, IRT is well-suited to the creation of a concise measure, since the reliability of IRT-derived measures do not increase with number of items [29]. Importantly, IRT approaches allow for the in-depth evaluation of whether each item is suitably difficult and able to discriminate between those with low and high ability. This evaluation of individual items could be invaluable in settings where a slightly different subset of items are administered to different individuals or groups (e.g., during adaptive testing, or for cross-cultural testing [33]). Owing to this item-level evaluation, IRT can better estimate a measure's precision than CTT, which relies upon single estimates such as Cronbach's α [34]. Accordingly, IRT is increasingly being recognised as a novel way to validate, modify, and condense existing scales, by determining which items provide us with reliable information about a latent construct [35]. In this way, IRT can determine which items in an existing measure should be removed, modified, or added to best represent all dimensions of the latent construct, increasing the validity of the overall scale [36].

### 1.4 The current research

In line with suggestions that IRT can helpfully supplement the strengths of CTT [37], the current study used IRT to develop a concise, and psychometrically grounded measure of environmental knowledge.

There are different approaches to IRT, with some previous measures having adopted the simplest IRT models, known as 1-parameter (1-PL) or Rasch models [16]. These models extend CTT by estimating the probability of an individual getting a correct response based on the item's difficulty level. Though invaluable in certain contexts, these models are based upon the restrictive assumption that for every test item, the highest ability participants will have a higher than 50% chance of correctly answering the question, and lowest ability participants lower than 50% chance [38]. Because of this, it is rare for a Rasch model to fit well for scales including more than just a few items [39]. As well as estimating item difficulty, the two-parameter (2-PL) IRT model includes a discrimination parameter, which permits item responses to be differentially related to the latent trait, meaning that items with higher discrimination values make better distinctions between respondents whose true scores lie above and below the item's difficulty level. As a result, evaluating a discrimination parameter adds flexibility and improves data fit.

Whilst some work has suggested that allowing item discrimination to vary may result in a test not having the same meaning for each test-taker [38], this is more relevant when administering a measure to determine differences between individuals, than during measure development. Indeed, in development, it is more important to be able to make accurate group level inferences about how well an item is performing across all participants, than to understand how well individual participants are performing across items. Discrimination values help to achieve this, making 2-PL IRT models an extremely powerful tool for questionnaire development, evaluation, and refinement, often resulting in concise and valid instruments [33].

Building upon the limitations of CTT, and fulfilling the need for a concise and psychometrically grounded measure of environmental knowledge, the current study will be the first to use IRT discrimination values to select the best performing items from an existing measure, and develop a short, robust measure. To this end, we assessed the factor structure of Geiger et al.’s Environmental Knowledge Test [19] in a British sample, used IRT to analyse each item's psychometric properties and reduce items, and re-examined the new measure's factor structure.

## 2 Materials and methods

### 2.1 Participants and materials

A UK sample of 346 undergraduate students were recruited via the University's online research participation scheme, and by advertising the study in undergraduate lectures (37 male, 308 female, 1 other; M_age_ = 19.06 years, SD_age_ = 2.05). Respondents received course credit for their participation. One hundred and twenty-one of these participants repeated the study nine weeks later for test-retest analyses. These figures do not include 43 additional participants who were excluded for either failing to complete the study or an attention check, in which participants were instructed to ‘select slightly agree’ to check they were paying sufficient attention to the questions. In the retest sample, six additional participants were excluded for failing the aforementioned attention check. Our variable-to-factor ratio was 12.1, exceeding the recommended value of six needed for adequate power in factor analyses [40]. Our sample size also exceeded the minimum standard of 250 participants for IRT models with 30 items [41].

Ethical approval was granted by the University's ethics committee (project code: 19–214). Participants gave informed consent and completed the study online using survey platform Qualtrics [42], which included an adapted version of Geiger et al.’s [19] 36-item Environmental Knowledge Test (EKT). All items were multiple choice with one correct and three distractor answers. Items spanned system knowledge (e.g., ‘What is the meaning of the abbreviation CO_2_?’), action-related knowledge (e.g., ‘Which energy form is a renewable form of energy?’) and effectiveness knowledge (e.g., ‘For which material does recycling save the most energy in comparison to new production?’). The scale was developed in German accompanied with an English translation; the latter was used in the present study. Here, we removed six culturally-specific items to make the measure suitable for non-German samples and made small modifications to improve item clarity and accuracy in a British sample (see Supplementary Materials: Table S1 for all items and modifications).

### 2.2 Data analysis

All data were analyzed in SPSS and R [43]. In SPSS, we computed descriptive statistics, interclass correlation coefficients (ICC) and correlations following re-test. In R, we conducted two nested confirmatory factor analyses (CFA), using the *lavaan* package [44]. The following indices were considered when assessing model fit [45]: (1) chi-square (χ^2^), for which non-significant scores indicate good fit; (2) Comparative Fit Index (CFI); (3) Tucker-Lewis Index (TLI), for which ≥ 0.90 indicate good fit; (3) Root Mean Square Error of Approximation (RMSEA), which should be < 0.08, and Standardized Root Mean Square Residual (SRMR), for which < 0.08 is considered a good fit. R was also used to analyse the psychometric properties of discrimination, thresholds, information curves for the individual items, and test the assumption of unidimensionality using the *ltm* package [46]. The *psych* package was used to determine omega and greatest lower bound (GLB) coefficients, two robust measures of internal consistency (see Ref. [47]). As data were binary, the robust Diagonally Weighted Least Squares (DWLS) was used, since it is specifically designed for binary data, and provides more accurate parameter estimates and robust model fit compared to the commonly used Maximum Likelihood (ML [48]). A dichotomous model was adopted for use with binary data. One-, two- and three-parameter logistic models (1-PL; 2-PL; 3-PL) were tested. Akaike Information Criterion (AIC), for which smaller numerical values indicate better model fit, were used to determine which logistic model was most suitable. We assessed difficulty levels using an item threshold analysis, which indicates the skill level required to achieve a 0.5 probability of a correct response. Whilst easier items tend to be endorsed by many individuals, harder items tend to only be endorsed by those with high environmental knowledge [49].

We used discrimination values, derived from item response theory, to remove the most problematic items. An item's discrimination refers to its ability to distinguish between individuals lower and higher in environmental knowledge, with higher values indicating higher ability to discriminate [49,50]. We performed a final CFA on the new, shortened scale, to demonstrate good model fit in line with theoretical literature.

## 3 Results

### 3.1 Confirmatory factor analysis

A nested CFA was performed to test whether the EKT reflected the factor structure theorised in the literature. The EKT had excellent model fit across several fit indices for the one- and three-factor models (see Table 1), with a chi-squared difference test showing no significant difference between the two models, Δχ^2^(3) = 0.92, *p* = .821.

**Table 1:** Model fit indices for the Environmental Knowledge Test.

Model	χ^2^(df)	CFI	TLI	RMSEA [90% CI]	SRMR
One-Factor	418.86 (405), *p* = .307	.95	.95	.01 [.00 - .02]	.095
Three-Factor	416.67 (402), *p* = .296	.95	.94	.01 [.00 - .02]	.095

### 3.2 Discrimination and thresholds

A dichotomous 2-PL logistic model was used to calculate the thresholds and discrimination of items (Akaike Information Criterion [AIC] = 11617.67). The assumption of approximate unidimensionality was confirmed using modified parallel analysis (*p* = .772 [51]). To confirm that a 2-PL best fit the data, a 1-PL model was also tested. Despite being more parsimonious, overall fit was poorer than the 2-PL model (AIC = 11825.81). A 3-PL model, including a guessing parameter, was tested, but model fit was also not improved (AIC = 11662.75). An item threshold analysis showed that 15 EKT items were easier than average (*b* < −1.5), with eight of appropriate difficulty (−1.5 < *b* < 1.5), and seven harder than average (*b* > 1.5) ([52]; Table 2).

**Table 2:** Item parameters for the Environmental Knowledge Test.

	Item	Type	Domain	*M*	*SD*	Discrimination *(a)*		Difficulty *(b)*	
*	1	S	Ecology	.71	.45	0.115	Very low	−7.835	Easier
	2	S	Ecology	.82	.39	0.820	Moderate	−2.070	Easier
*	3	S	Ecology	.05	.23	0.184	Very low	15.577	Harder
	4	S	Ecology	.74	.44	0.739	Moderate	−1.554	Easier
	5	S	Ecology	.71	.46	0.455	Low	−2.037	Easier
	6	S	Climate	.88	.33	0.809	Moderate	−2.697	Easier
	7	S	Climate	.98	.15	1.708	Very high	−2.944	Easier
	8	A	Climate	.75	.43	1.021	Moderate	−1.284	Average
	9	S	Climate	.78	.42	0.364	Low	−3.587	Easier
	10	A	Climate	.78	.41	0.790	Moderate	−1.830	Easier
*	11	S	Resources	.27	.44	−0.159	Negative	−6.425	Easier
	12	E	Resources	.33	.47	0.426	Low	1.770	Harder
*	13	E	Resources	.29	.46	0.137	Very low	6.505	Harder
	14	S	Consumption	.80	.40	1.071	Moderate	−1.554	Easier
	15	A	Consumption	.87	.34	1.368	High	−1.789	Easier
*	16	A	Consumption	.46	.50	0.341	Very low	0.524	Average
	17	E	Consumption	.70	.46	0.567	Low	−1.597	Easier
*	18	E	Consumption	.47	.50	0.295	Very low	0.360	Average
*	19	E	Consumption	.34	.48	−0.051	Negative	−13.032	Easier
*	20	E	Consumption	.49	.50	0.008	Very low	6.076	Harder
*	21	E	Consumption	.42	.49	0.033	Very low	10.023	Harder
	22	E	Society & Politics	.67	.47	0.446	Low	−1.665	Easier
	23	S	Society & Politics	.78	.41	1.078	Moderate	−1.451	Average
	24	S	Society & Politics	.38	.49	0.795	Moderate	0.709	Average
	25	S	Economy	.61	.49	0.749	Moderate	−0.670	Average
	26	S	Economy	.62	.49	0.944	Moderate	−0.591	Average
	27	S	Contamination	.69	.46	0.550	Low	−1.587	Easier
*	28	A	Contamination	.25	.43	0.138	Very low	7.965	Harder
	29	A	Contamination	.51	.50	0.587	Low	−0.106	Average
*	30	S	Contamination	.41	.49	0.044	Very low	8.155	Harder
	System			63.88%	2.22				
	Action-Related			60.33%	1.21				
	Effectiveness			46.38%	1.38				
	Total			58.5%	3.49				

*Note*. Items marked with (*) were removed. Knowledge types are denoted with S (System), A (Action-Related) and E (Effectiveness). See Table S1 for questions.

Table 2 presents the discrimination values and classifications for the EKT items. Using Baker's classification [49], one item had very high discrimination (*a* > 1.7), one item was high (1.35 < *a* < 1.69), 10 items were moderate (0.65 < *a* < 1.34), seven items were low (0.35 < *a* < 0.64) and nine items were very low (0.01 < *a* < 0.34). Two items had negative discrimination, indicating that the higher someone's environmental knowledge, the lower they tended to score on these items. These were deemed poorly-conceived items and discarded.

We removed all items which were very low or negatively discriminating (see Table 2), which concurrently removed the EKT's most difficult and easiest items.

### 3.3 Item information curves

**Fig. 1:**
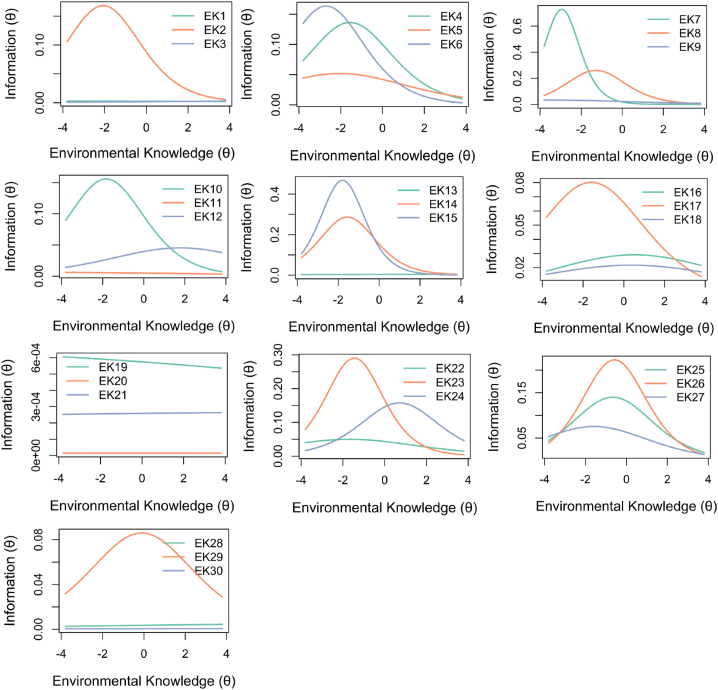
Item information curves for all Environmental Knowledge Test items.

We additionally inspected the Item Information Curves (IIC; Fig. 1), showing how much information each item shares with the overall measure [53]. This indicates the value for which an item is best at measuring the respondent's environmental knowledge, with steeper curves indicating a more informative item. More informative items, shown as steeper curves, are typically higher in measurement precision and lower in measurement error, indicating higher reliability [52]. In 2-PL models, higher information is determined by higher item discrimination and difficulty at different levels of environmental knowledge, relative to other items [54]. Accordingly, removing the lowest discriminating items generally removed the least informative items.

Though slightly more discriminating than the removed items, items nine, 22, and 27 were also not particularly informative, and were easier than average. However, these were retained to ensure at least 1–2 items addressed each environmental domain and type of environmental knowledge (see Supplementary Materials: Table S1) . Item nine was retained as it was the only item addressing the consequences of climate change. This approach, of balancing conceptual validity and psychometric robustness, was followed in line with Graham et al. [55]. Therefore, item analysis was psychometrically-grounded whilst ensuring the integrity of the latent construct, which ultimately resulted in a shortened, 19-Item Environmental Knowledge Test (EKT-19).

### 3.4 Final EKT-19 items

After the removal of 11 items, six of the remaining items were of optimal difficulty (−1.5 < *b* < 1.5 [52]), one was slightly harder (item 12; *b* = 1.77), and twelve were slightly easier (−3.59 < *b* < −1.55). However, all the easiest and most difficult items were removed. The final 19 items consisted of 12 system items, four action-related items, and three effectiveness items. This is in line with recommendations that each factor should contain at least three items [56], and the split of items is relatively similar to Geiger et al. [19], who started with 21 system items, seven action-related, and eight effectiveness items. Similarly, at least one item was retained from each environmental domain, ensuring that all dimensions of environmental knowledge can be fairly assessed (Ecology: items 2, 4, 5; Climate: 6, 7, 8, 9, 10; Resources: 12; Consumption Behaviours: 14, 15, 17; Society and Politics: 22, 23, 24; Economy: 25, 26; Environmental Contamination: 27, 29).

### 3.5 Confirmatory factor analysis of the EKT-19

To confirm that the EKT-19 retained the same one- and three-factor structure theorised in the literature and the 30-item measure, we conducted two final nested CFAs. The EKT-19 revealed excellent and improved model fit across both the one- and three-factor solutions compared to the original measure (Table 3), with no difference in goodness-of-fit between models, Δχ^2^(3) = 1.12, *p* = .773. Whilst we could not statistically compare the EKT and EKT-19 owing to their non-nested structure and different variables, the EKT-19 demonstrated improved CFI, TLI, RMSEA and SRMR compared to the EKT, indicating the EKT-19 is a more concise, discriminative, and improved measure.

**Table 3:** Model fit indices for the EKT-19.

Model	χ^2^(df)	CFI	TLI	RMSEA [90% CI]	SRMR
One-Factor	147.35 (152), *p* = .591	1.00	1.02	.00 [.00 - .02]	.080
Three-Factor	145.51 (149), *p* = .566	1.00	1.01	.00 [.00 - .02]	.080

### 3.6 Reliability and practical utility of the EKT-19

Internal consistency of the measure was moderate-to-good (ω = 0.67; GLB = 0.74), particularly given that the EKT-19 captures multiple components of environmental knowledge. Test-retest reliability was determined using 121 participants, who completed the EKT at two time points separated by nine weeks. Scores were highly correlated between timepoints (*r* = 0.76, *p* < .001) and intra-class correlations (ICC; α = 0.87) indicated excellent test-retest reliability [57]. In line with previous scale development [21], we examined whether the EKT-19 saved time compared to the EKT. The average completion time per item was 15.37 s, meaning that, on average, the EKT took 7.7 min to complete. In comparison, the EKT-19 took approximately 4.9 min to complete, meaning that the EKT-19 was almost 3 min faster per participant. If researchers wished to pay their participants the living wage (GBP 9.50 per hour), the EKT-19 saves GBP 0.44 per participant, compared to the EKT.

## 4 Discussion

With rising impetus to understand the contribution of environmental knowledge to climate-related outcomes, we used IRT to develop a short, reliable, and psychometrically-grounded measure of environmental knowledge (EKT-19). First, we showed that the original EKT reflected the theorised unidimensional structure in a UK sample, providing the first validation that this measure maintains its structure in non-German samples. Importantly however, we also find a good three-factor fit, congruent with theories that environmental knowledge can be separated into knowledge types [12]. Given this finding, we tentatively suggest that theoretical and empirical debate surrounding the uni-vs multi-dimensionality of environmental knowledge may be unwarranted. Indeed, environmental knowledge may be conceptualised as an overall construct, comprised of three distinct knowledge types: system, action, and effectiveness [13].

For the first time, we used discrimination values derived from IRT to determine which EKT items were most able to determine one's environmental knowledge. Such approaches are rare in environmental psychology and have only recently been used to successfully refine longstanding measures such as the New Ecological Paradigm (NEP [31]; see Ref. [32]). Guided by similar approaches in health research (e.g., Ref. [35]), our study is the first to use discrimination values to determine which items in an existing measure did not yield reliable information about a person's environmental knowledge, and should be removed. Using discrimination values, we extend Geiger et al.’s findings [19], who speculated that specific items (11, 16, 18, 20, and 30) may have contributed to their reduced model fit. Three of these items (16, 18, 20), related to consumption behaviours, one item (11) to resources, and one item (30) to environmental contamination. We confirmed that, even after making slight changes to wording for clarity in a British sample, these items were poorly discriminating, perhaps due to unclear wording for some items (e.g., 11 and 16; see Table S1). After removing the most poorly discriminating items, we found excellent one- and three-factor model fits with moderate-to-good internal reliability, and excellent test-retest reliability.

In line with our suggestion that environmental knowledge can be conceptualised as both a one- and three-factor structure, we propose that the EKT-19 may be used as a reliable measure of both overall and sub-types of environmental knowledge. Such conceptualisations are common when measuring clinical constructs (e.g., Autism-Spectrum Quotient [24]), which have had a longer tradition of psychometric development, and have commonly contributed to the theoretical understanding of disorders (e.g., sub-clinical symptoms of Autism). Though some environmental research has used both subscale and total scores (e.g., of pro-environmental behaviours [58]), research has rarely tested the psychometric validity of the overall and subscale scores within the same measure as we have. Given the challenges caused by different questions being used to conceptualise environmental knowledge in previous work, the EKT-19 could provide much needed consistency when measuring all forms of environmental knowledge, give confidence in future work investigating its correlates, and contribute to our theoretical understanding of environmental knowledge. Importantly, the current item-level analysis provides assurance of the validity of individual items, which can be particularly beneficial when researchers only wish to use specific items. Such approaches could be particularly beneficial in computer adaptive testing, which adapt the questions given to each participant depending on ability level [59].

The moderate-to-good internal consistency of the EKT-19 reflects the need to achieve sufficient internal consistency, whilst maintaining confidence that we represent environmental knowledge in full. Indeed, Graham et al. [55] argued that it is preferable to retain dissimilar, moderately correlated items than to select redundant, similar items that do not comprehensively capture various facets of the construct. For example, though item nine (‘which natural phenomenon is not attributed to climate change?’) is less informative than other items, its inclusion is vital to understanding knowledge about the consequences of climate change. In contrast to many other environmental knowledge measures [13,15], the EKT-19 is considerably shorter, making it more engaging. Long measures can be problematic in several ways, especially in cognitively demanding knowledge tests [22]. For example, results may be compromised by participant fatigue, poor attention, or boredom. Specifically, approximately 3 min per participant would be saved using the EKT-19 compared to the EKT, thus saving valuable time, and minimising data quality concerns.

Beyond the conceptual and practical implications of this study, our findings have potential implications for the design of educational programmes. Our excellent one-factor fit suggests that system, action-related and effectiveness knowledge somewhat co-occur, inferring that system-level knowledge may boost one's understanding of which actions are most environmentally-friendly, and how effective they are, or vice versa. This finding indicates the value of teaching about all three types of knowledge in educational interventions to promote pro-environmental outcomes. Future research may further explore this by determining if, when measured robustly using the EKT-19, the different types of knowledge differentially predict pro-environmental behaviour (e.g., if action-related and effectiveness knowledge are better predictors than system knowledge). Further, consistent with previous research [13], we find that whilst participants scored similarly on system and action-related knowledge types, effectiveness knowledge was slightly lower. Considering that effectiveness knowledge is thought to directly predict pro-environmental behaviour [13], educational interventions may facilitate environmental outcomes by enhancing effectiveness knowledge.

Though we usefully selected the best items of the EKT, our research highlights the need to develop items that further discriminate the very highest ability test takers. When developing such items, questions must be sufficiently ‘difficult’ to differentiate those with high and low environmental knowledge but should not be difficult just because they are ambiguous to all test takers. This was seen in several of the removed EKT items (e.g., items 28 and 30 about environmental contaminants). Relatedly, the current work indicates the need to develop more questions of optimal difficulty. Indeed, whilst our analysis removed the easiest and hardest items, we retained several items which were deemed slightly too easy, to ensure there was at least one item in each environmental domain and adhere to recommendations to retain at least three items per factor [56]. Since questions were originally taken from older measures (e.g., Refs. [16,20]), people may be generally more knowledgeable about environmental issues today than ever before. This is especially the case with growing sustainability-related media coverage [60], and increased environmental awareness driven by the Covid-19 pandemic [61]. Accordingly, it is possible that questions designed two decades ago are not well suited to current ability levels, and harder questions must be developed. To address the need for more discriminative and difficult questions, future research may adopt a Delphi technique, to gather expert opinions about relevant environmental issues and guide the conception of new items [62].

Alongside identifying the need for further item development, the current analysis helps guide which items require it. For example, of the remaining effectiveness knowledge items, all three items (12, 17, 22) are less discriminating, and further from optimum difficulty, than other items. This contrasts with items measuring action-related and system knowledge, which are generally better discriminators and closer to the optimum difficulty level. Accordingly, the current analysis indicates a need for the development of new effectiveness items, perhaps using a bottom-up Delphi approach. Similarly, the current item-level analysis shows that some environmental domains have more appropriate items than others. For example, the two remaining items assessing environmental contaminant knowledge have relatively low discriminative properties compared to other items, and difficulty slightly harder than desired, and so may require modifications. The current work therefore not only informs which items cannot reliably assess environmental knowledge and thus were removed, but also which ones could be further improved.

An alternative explanation for some items appearing slightly easier than desired is that our student sample was more knowledgeable about environmental issues than the general population would be. Though this assumption has been made in previous research [16], we observed slightly lower knowledge in our student sample (58.5%) compared to non-student samples (68.6%, [19]; 64.6% [63]), perhaps indicating that students’ environmental knowledge is not superior to people in the general population. Nonetheless, future research may advance the current work by testing the EKT-19 in more representative, general population samples. Such work should seek to recruit a more balanced sex distribution, to allow for an analysis of measurement invariance across sex and determine if the EKT-19 is invariant between males and females (see Ref. [64] for recent discussion). Considering the current sample, future work may also administer the EKT-19 across diverse cultural groups, to determine if our removal of culturally-specific items was sufficient to maintain relevance cross-culturally, or if new questions that are generalisable cross-culturally need to be developed. In the situation that certain items do not perform as well as others in certain cultures, the current IRT-led approach will be beneficial, since it will be possible to choose and drop questions as appropriate for that population. Further, given that environmental knowledge was highly correlated with general knowledge in the original EKT, future work should demonstrate discriminative validity by comparing the EKT-19 with a similar, yet distinguishable construct such as environmental awareness. Finally, the current work may be extended by determining the predictive validity of the EKT-19, by testing its relationship with related constructs such as pro-environmental behaviours. This is especially the case considering that little previous work has explored the predictive properties of environmental knowledge measures (e.g., Ref. [13]). Similarly, Geiger et al. [19] did not explicitly test if the original EKT reliably predicted pro-environmental behaviours. Instead, they assumed that environmental knowledge was represented by general knowledge, and tested the relationship between general knowledge and pro-environmental behaviours. However, given that environmental knowledge is often a weak direct predictor of climate-related outcomes such as pro-environmental behaviour [65], future work should carefully consider the best way to test the predictive validity of the EKT-19, perhaps also considering the moderating impact of environmental values and attitudes [17].

In summary, we sought to build upon the CTT approach of Geiger et al. [19], and present a short, psychometrically robust measure of environmental knowledge (EKT-19). For the first time, we validated the factor structure of an existing measure in a British sample, used item-level discrimination scores to evaluate its psychometric properties, and determined which items were most able to differentiate between participants with different levels of environmental knowledge. After removing the lowest discriminating items, we find moderate-to-good internal consistency, excellent test-retest reliability, and excellent one- and three-factor fit in line with theoretical and empirical literature. Alongside selecting the best items, the current analysis helpfully informed which items may require further bottom-up development. Overall, we suggest that the EKT-19 is a concise, conceptually robust, and reliable measure of overall and sub-scaled environmental knowledge.

## Funding statement

This work was supported by a scholarship awarded to Lois Player from the EPSRC Centre for Doctoral Training in Advanced Automotive Propulsion Systems [project code: EP/S023364/1].

## Author contribution statement

Lois Player: Conceived and designed the experiments; Performed the experiments; Analyzed and interpreted the data; Contributed reagents, materials, analysis tools or data; Wrote the paper. Paul H. P. Hanel: Conceived and designed the experiments; Performed the experiments; Analyzed and interpreted the data; Wrote the paper. Lorraine Whitmarsh: Conceived and designed the experiments; Wrote the paper. Punit Shah: Conceived and designed the experiments; Performed the experiments; Contributed reagents, materials, analysis tools or data; Wrote the paper.

## Data availability statement

Data associated with this study has been deposited on the University of Bath Research Data Archive (https://doi.org/10.15125/BATH-01204).

## Declaration of competing interest

The authors declare that they have no known competing financial interests or personal relationships that could have appeared to influence the work reported in this paper.
