# Supplementary material for: The 19-Item Environmental Knowledge Test (EKT-19): A short, psychometrically robust measure of environmental knowledge
Source: Heliyon. 2023 Jun 30;9(8):e17862. doi: 10.1016/j.heliyon.2023.e17862 (PMC10440470; doi:10.1016/j.heliyon.2023.e17862)
Supplement: Multimedia component 1 [file mmc1.docx]

**Supplementary Materials**

**Table S1**

*EKT Questions in the Current Study*

| **Type** | **Domain** | **N^o^** | **Item** | **Answers (correct in bold)** |
| --- | --- | --- | --- | --- |
| *S | Ecology | 1 | Which statement is correct? | 1. **Groundwater is composed of surface water that seeps into the ground (rain, rivers, lakes)** 2. Groundwater comes from deep geological layers 3. Pollutants from river and rainwater do not get to the groundwater 4. Groundwater is old and is no longer being formed |
| S | Ecology | 2 | What causes wind? | 1. The movement of the clouds 2. **Differences in temperature and air pressure in the atmosphere** 3. The moon’s gravity 4. Ocean currents |
| *S | Ecology | 3 | On average, how long does it take to form 10cm of fertile soil? | 1. 10 years 2. 80 years 3. 500 years 4. **2000 years** |
| S | Ecology | 4 | What is an ecological niche? | 1. The place where an animal species lives the longest throughout the year 2. **The interaction of biotic and abiotic nature in a specific place** 3. The place where animals return to have their offspring 4. The place where prey species typically hide |
| S | Ecology | 5 | Which of the following aspects is not a part of biodiversity? | 1. **Demographic diversity** 2. Diversity of species 3. Diversity of ecosystems 4. Genetic diversity |
| S | Climate | 6 | Which of the following phenomena has been the main cause of *climate change* over the last 20 years? [Original wording: global warming] | 1. Reduction of the ozone layer (the so-called ozone hole) 2. **Increased emissions of greenhouse gases (the so-called greenhouse effect)** 3. Changes in ocean currents, e.g., “El Niño”. 4. Changes in the skewness of the earth’s axis |
| S | Climate | 7 | What is the meaning of the abbreviation CO_2_? | 1. Carbon monoxide 2. Greenhouse effect 3. **Carbon dioxide** 4. Climate change |
| A | Climate | 8 | Which energy form is a renewable form of energy? | 1. Nuclear energy *(from fission)* 2. Petroleum 3. Natural gas 4. **Geothermal energy** |
| S | Climate | 9 | Which natural phenomenon is not attributed to climate change? | 1. Glacial melting 2. Sea level rise 3. Change in ocean currents 4. **Earthquakes** |
| A | Climate | 10 | Which action does not help reduce the greenhouse effect? | 1. Heating a house with solar heat 2. ***Removing the catalytic converter from your vehicle*** [Original item: Driving a car with a catalyser] 3. Saving energy in everyday life 4. Planting trees |
| *S | Resources | 11 | International environmental experts have identified 9 factors that are crucial for the Earth’s system stability. Which factor’s limit has been exceeded? | 1. **Climate change** 2. Freshwater consumption 3. Ocean acidification 4. Ozone depletion in the stratosphere (ozone hole) |
| E | Resources | 12 | For which material does recycling save the most energy in comparison to new production? | 1. **Aluminium** 2. Glass 3. Tinplate 4. Paper |
| *E | Resources | 13 | Which food requires the most water for production? | 1. **1kg coffee** 2. 1kg rice 3. 1kg potatoes 4. 1kg apples |
| S | Consumption Behaviour | 14 | What was the original principle of sustainability? | 1. Resources must be distributed fairly 2. Resources must be used an environmentally friendly manner 3. **Resources must not be used at a higher rate than they can reproduce naturally** 4. Resources have to be deployed efficiently |
| A | Consumption Behaviour | 15 | What does the carbon footprint of a product mean? | 1. The typical staining of the sky that is caused by high CO2 concentrations 2. **The amount of all greenhouse gas emissions that is emitted over the lifecycle of a product** 3. The amount of CO2 a product emits when it corrodes 4. The chemical change that is caused by Co2 in the atmosphere |
| *A | Consumption Behaviour | 16 | Which action does not help to save energy costs in everyday life? | 1. **Leaving the windows *slightly ajar instead of opening it completely for a short time, when the heating is on*** [Original wording: tilted in the heating season instead of opening the window completely for a short time] 2. Insulating buildings properly, specifically roofs and windows 3. Keeping freezers free of ice 4. Providing washing machines with a separate warm water supply |
| E | Consumption Behaviour | 17 | Which type of transport produces the least amount of emissions per passenger and kilometre in short distance traffic? *(Please assume car, bus and motorcycle are fossil-fuel powered)* | 1. **Tram, subway and train** 2. Car 3. Bus 4. Motorcycle |
| *E | Consumption Behaviour | 18 | Which illuminant has the highest energy efficiency (the highest luman/watt)? | 1. Conventional light bulb 2. Halogen lamp 3. **LED (light emitting diode)** 4. Energy-saving light bulb |
| *E | Consumption Behaviour | 19 | Which of the following beverage packaging is the most harmful the environment? | 1. Reusable glass bottle 2. Reusable plastic bottle 3. Beverage carton (Tetrapak) 4. **Beverage can** |
| *E | Consumption Behaviour | 20 | An average household consumes the most energy for… | 1. Illumination 2. Warm water 3. **Heating, *ventilation and air conditioning (HVAC)*** 4. Electric devices |
| *E | Consumption Behaviour | 21 | In which of the following domains is the most water consumed in everyday life? | 1. **Flushing the toilet** 2. Eating and drinking 3. Washing dishes 4. Doing laundry |
| E | Society & Politics | 22 | *For the same amount of calories*, meat is … | 1. Half as pollutive as vegetables 2. As pollutive as vegetables 3. Twice as pollutive as vegetables 4. **Ten times more pollutive as vegetables** |
| S | Society & Politics | 23 | Which organisation is not mainly concerned with environmental issues? | 1. **WHO (World Health Organisation)** 2. Greenpeace 3. WWF (World Wildlife Fund) 4. FOEI (Friends of the Earth International) |
| S | Society & Politics | 24 | What is the Kyoto Protocol? | 1. A Japanese law on renewable energies. 2. **The first compulsory regulation of greenhouse gas emission in industrialized countries.** 3. An international protocol concerning the consequential damages of the Fukushima reactor disaster. 4. The first worldwide protocol for the regulation of emission trading valid for Japan. |
| S | Economy | 25 | What does “Greenwashing” *mean*? | 1. Environmentally-friendly washing cycles in modern laundry machines. 2. **Falsely conveying the impression of being environmentally-friendly.** 3. Environmentally-friendly production guidelines in the textile industry. 4. Environmentally-friendly removal of graffiti. |
| S | Economy | 26 | Which of the following are included in "corporate social responsibility" (CSR)? | 1. **Voluntary sustainability measures of companies** 2. Internal social security systems of companies 3. A label for particularly social companies 4. A business tax that can only be used for social purposes |
| S | Environmental Contamination | 27 | What is the main pollutant of ground water? | 1. **Agricultural fertilisers (e.g., with nitrates)** 2. Increasing car traffic 3. Industrial air pollution 4. Discharge of wastewater into the water bodies |
| *A | Environmental Contamination | 28 | Which item has to be disposed as hazardous waste? | 1. Old CDs/ DVDs 2. Foamed up Styrofoam packaging 3. **Used energy-saving light bulbs** 4. Empty spray cans |
| A | Environmental Contamination | 29 | Detergents and cleansers should be used as sparsely as possible, because… | 1. Intense foaming reduces washing performance 2. They contain high concentrations of heavy metals 3. Sewer pipes are affected 4. **The extraction of these substances in purification plants is difficult** |
| *S | Environmental Contamination | 30 | The world’s biggest continuous floating garbage patch (“the great ocean garbage patch”) is located in which ocean? | 1. **North Pacific** 2. Indian Ocean 3. North Atlantic 4. Southern Ocean |

*Note.* Type of knowledge, as conceptualised by Frick et al. (2004) and used by Geiger et al. (2019). System (S), Action (A), Effectiveness (E). Edits to original wording in *italics*. Original wording provided in [ ] where required. Asterisk (*) denotes removed item based on discrimination value.
